# Privacy-preserving federated unsupervised domain adaptation with application to age prediction from DNA methylation data

**DOI:** 10.1093/bioinformatics/btaf465

**Published:** 2025-08-22

**Authors:** Cem Ata Baykara, Ali Burak Ünal, Nico Pfeifer, Mete Akgün

**Affiliations:** Medical Data Privacy and Privacy-Preserving Machine Learning, University of Tübingen, 72076 Tübingen, Germany; Medical Data Privacy and Privacy-Preserving Machine Learning, University of Tübingen, 72076 Tübingen, Germany; Institute for Bioinformatics and Medical Informatics, University of Tübingen, 72076 Tübingen, Germany; Institute for Bioinformatics and Medical Informatics, University of Tübingen, 72076 Tübingen, Germany; Medical Data Privacy and Privacy-Preserving Machine Learning, University of Tübingen, 72076 Tübingen, Germany; Institute for Bioinformatics and Medical Informatics, University of Tübingen, 72076 Tübingen, Germany

## Abstract

**Motivation:**

Generalizing machine learning models across small, high-dimensional, and heterogeneous biological datasets remains a critical challenge due to domain shifts caused by variations in data collection, population differences, and privacy constraints that restrict data sharing. Existing federated domain adaptation (FDA) approaches primarily rely on deep learning and focus on classification tasks, making them unsuitable for privacy-sensitive, small-scale regression problems in biomedical research. We introduce a privacy-preserving federated method for unsupervised domain adaptation in regression, enabling robust learning across distributed, high-dimensional datasets while maintaining full data privacy.

**Results:**

Our method is the first to enable distributed training of Gaussian processes for domain adaptation, ensuring complete privacy through randomized encoding and secure aggregation. Unlike deep learning-based FDA approaches, our method is specifically designed for small-scale, high-dimensional biological data, overcoming prior limitations in scalability and generalization. We evaluate our approach on age prediction from DNA methylation data, demonstrating that it achieves performance comparable to non-private state-of-the-art methods while fully preserving data privacy. This work enables secure and effective cross-institutional collaboration in biomedical research without requiring raw data sharing.

**Availability and implementation:**

The source code for our method is available at https://github.com/mdppml/FREDA.

## 1 Introduction

Machine learning (ML) has rapidly become a powerful tool with applications across numerous fields, including computational biology and healthcare, where it has shown great potential in solving complex problems (Valentini *et al.* 2009, Angermueller *et al.* 2016, Greener *et al.* 2022, Thumuluri *et al.* 2022). However, collecting and labeling biological datasets is often challenging, costly, and time-consuming. As a result, many datasets in these fields are small-scale, unlabeled, and heterogeneous, often collected from different sources under varying environmental and experimental conditions—such as different laboratories, hospitals, or institutions (Orouji *et al.* 2024). These challenges introduce two critical issues: (1) data from different sources often exhibit distinct statistical distributions while lacking labeled samples, complicating direct model transfer; and (2) privacy regulations and the sensitive nature of biomedical data often restrict data sharing across institutions, necessitating collaborative learning approaches.

Unsupervised federated domain adaptation (FDA) addresses these challenges by collaboratively aligning distributions between training and test data, referred to as source and target domains, respectively, without requiring direct data sharing (Farahani *et al.* 2021). The primary motivation for unsupervised FDA is the scarcity of labeled data in the target domain, making it impractical to train models from scratch. Most existing FDA methods aim to mitigate distributional differences between domains (Sun *et al.* 2016, Peng *et al.* 2019, Liang *et al.* 2022, Liu *et al.* 2024). While deep learning-based FDA methods have achieved success in computer vision (Ganin and Lempitsky 2015, Long *et al.* 2016, Sener *et al.* 2016, Feng *et al.* 2021), their application to biological data remains limited due to high dimensionality and small sample sizes. Moreover, FDA research has predominantly focused on classification tasks, while regression-based approaches remain significantly underexplored despite their importance in biomedical applications (Lundberg *et al.* 2018, Poplin *et al.* 2018, Li *et al.* 2022).

In the broader domain adaptation literature, most advances have focused on image classification, where abundant labeled data and low-dimensional features facilitate domain transfer (Wang and Deng 2018, Weng *et al.* 2023, Yue *et al.* 2023).Approaches such as Domain-Adversarial Neural Networks (Ganin *et al.* 2016) and Maximum Mean Discrepancy-based methods like Deep Adaptation Networks (Long *et al.* 2015) align feature representations across domains, while self-supervised methods like CDAN (Long *et al.* 2018) integrate task-specific predictions to improve adaptation. These methods perform well on image benchmarks but are less suited to high-dimensional, small-sample biological datasets.

Domain adaptation for regression remains even less explored, as aligning continuous output spaces is particularly challenging. For instance, DINO (Wu *et al.* 2022) leverages distribution-aware neural networks to mitigate distribution shifts in regression tasks, achieving strong performance in image-based settings with large datasets. However, it remains untested on high-dimensional, low-sample-size biological data. A key method for unsupervised adaptation in this setting is WENDA (Handl *et al.* 2019), which estimates feature dependencies and applies adaptive regularization to handle domain shifts. WENDA has been shown to perform well on DNA methylation-based age prediction, making it a relevant baseline for our work.

Federated settings further complicate domain adaptation due to both data heterogeneity and privacy constraints. Methods such as PartialFed (Sun *et al.* 2021) dynamically mix global and local model parameters, improving performance on cross-domain classification tasks. Similarly, FedGP (Jiang *et al.* 2024) enhances adaptation in low-data scenarios by filtering noisy gradients and optimally combining source and target information. However, these approaches focus on image datasets with abundant samples and low-dimensional features, making their applicability to high-dimensional biological data uncertain.

In this context, we introduce FREDA (**f**ede**r**at**e**d **d**omain **a**daptation), a novel method for privacy-preserving, federated unsupervised domain adaptation in regression tasks. Unlike conventional deep learning-based approaches that struggle with data scarcity and high dimensionality, FREDA is the first method to leverage distributed training of Gaussian processes regressors (GPRs), enabling collaborating entities to model complex features without pooling their private data.

Gaussian processes are particularly well-suited for feature modeling due to their probabilistic nature, providing not only point predictions but also uncertainty estimates in the form of Gaussian-distributed confidence intervals. This property is especially valuable in domain adaptation, where assessing prediction reliability is crucial when transferring knowledge across domains. However, like other kernel-based algorithms, GPRs require pairwise computation of data matrices, making it extremely challenging to train them when data is distributed across entities that cannot share raw samples.

To overcome this, FREDA introduces a novel combination of randomized encoding and secure aggregation, enabling distributed training of Gaussian processes while preserving complete data privacy. By facilitating robust feature modeling without direct access to raw data, FREDA is particularly well-suited for biological datasets, where privacy constraints, limited sample sizes, and data heterogeneity pose significant challenges.

We evaluate FREDA on a challenging benchmark task of age prediction from DNA methylation data. Our results demonstrate that FREDA achieves performance comparable to non-private methods while preserving complete data privacy. By addressing the challenges of small-scale, heterogeneous, and privacy-sensitive regression problems, our approach significantly expands the applicability of domain adaptation to real-world biomedical applications. Our contributions are as follows:

- We propose FREDA, the first method to enable privacy-preserving, federated unsupervised domain adaptation for regression tasks, specifically designed for small-scale, high-dimensional biological datasets.
- Through a novel combination of randomized encoding and secure aggregation techniques, FREDA is the first method to enable the distributed training of GPRs for effective feature modeling while ensuring complete data privacy.
- We evaluate FREDA on the challenging task of age prediction from DNA methylation data, demonstrating that it effectively models complex feature relationships in small-scale, heterogeneous, and distributed biological datasets, achieving performance comparable to non-private approaches while preserving privacy.

## 2 Related work

Unsupervised domain adaptation has been widely studied, primarily in image classification, where abundant labeled data and low-dimensional features facilitate domain transfer (Wang and Deng 2018, Weng *et al.* 2023, Yue *et al.* 2023). Many approaches focus on aligning feature representations across domains using adversarial training, such as Domain-Adversarial Neural Networks (Ganin *et al.* 2016) and Maximum Mean Discrepancy-based methods like Deep Adaptation Networks (Long *et al.* 2015). Self-supervised learning techniques, including CDAN (Long *et al.* 2018), further integrate task-specific predictions to improve adaptation. While these methods perform well on image benchmarks, they are less suited to high-dimensional, small-sample biological datasets.

## 3 Materials and methods

We consider the following distributed setting: there are *N* source domain clients, each with a local labeled dataset $X^{s_{i}}={\left\{(x_{j}^{s_{i}},y_{j}^{s_{i}})\right\}}_{j=1}^{n_{i}}$ and a sample size of $n_{i}$. The entire source domain data, distributed across the *N* clients, is denoted as $X^{S}=\overset{N}{\underset{i=1}{\cup }}X^{s_{i}}$. Similarly, there is a target client with a dataset $X^{T}={\left\{x_{m}^{t}\right\}}_{m=1}^{n_{t}}$ containing $n_{t}$ samples for the same prediction task, but without any available labels. In both the source and target domain datasets, the samples $\left\{x_{j}^{s_{i}}\right\}$ and $\left\{x_{m}^{t}\right\}$ are P-dimensional vectors, where P∈N, and the labels $\left\{y_{j}^{s_{i}}\right\}$ are scalars. The goal is to leverage source domain data to train a model that generalizes well to the target domain without explicit data sharing.

**Figure 1.**
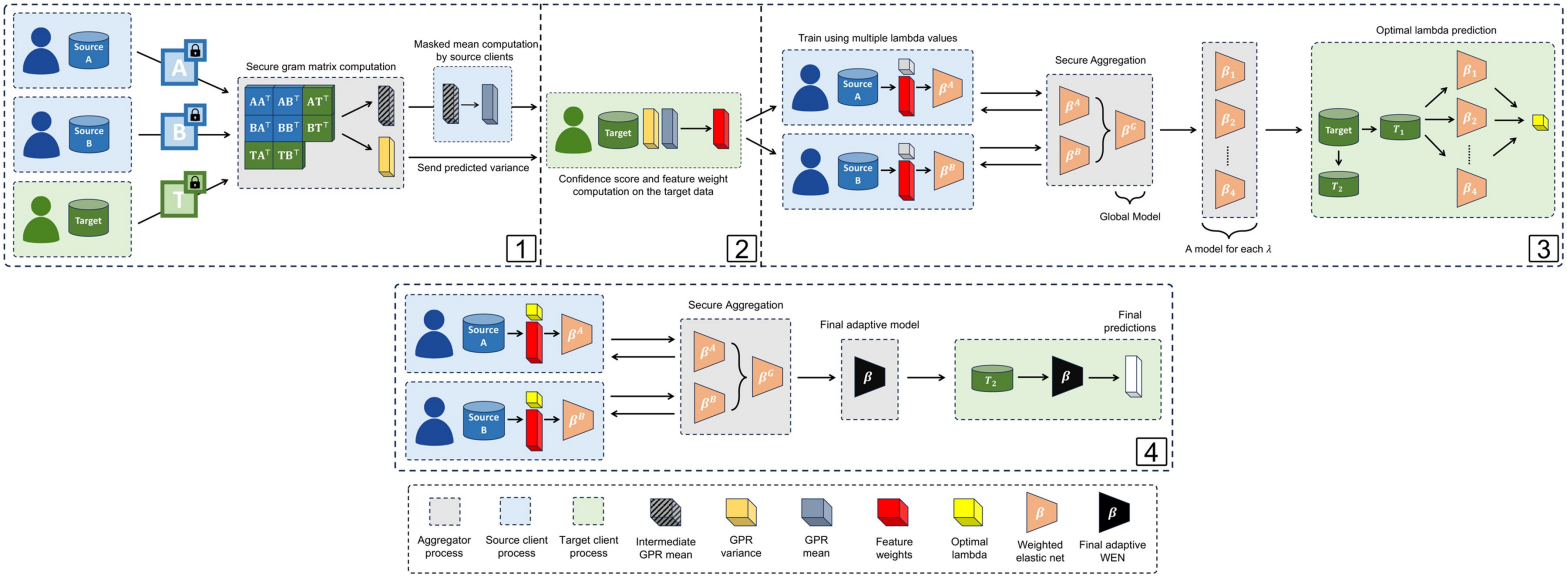
Overview of the FREDA framework for unsupervised domain adaptation in a federated setting. The framework consists of four phases: (1) Feature Model Training, where each feature is modeled using federated hyper-parameter optimization and GPRs with secure aggregation; (2) Feature Weight Computation, where the target client computes confidence scores from the predicted feature distributions and converts them into weights, which are shared with source clients via an aggregator; (3) Optimal Lambda Prediction, where multiple weighted elastic nets with varying regularization parameters (λ) are trained, and the optimal λ values are selected by the target client and shared with all participants; and (4) Final Adaptive Model Training, where the final adaptive WEN models are trained federatively and sent to the target client for inference.

FREDA follows four key steps (see Fig. 1): (1) learning feature dependencies via federated feature models, (2) computing confidence scores to derive feature weights, (3) predicting optimal regularization parameters through federated training, and (4) training the final adaptive model. While we describe the method for a single target domain, FREDA can be extended to multiple target domains as demonstrated in Section 4. A high-level summary of the algorithm as well as a detailed security analysis of the protocol, including communication assumptions and privacy guarantees under a semi-honest model, is provided in the Supplementary Material, available as supplementary data at *Bioinformatics* online.

### 3.1 Feature model training

Following the motivation of Handl *et al.* (2019), we begin by modeling dependencies between features. In the absence of target labels, this approach allows us to estimate how well each feature can be explained by the others, based on patterns learned from the source domain. The intuition is that features with stable dependency structures across domains are more likely to generalize and should be weighted more heavily in the final regression model.

In our distributed setting, we must compute these feature dependencies without sharing raw data between parties. To achieve this, we leverage Bayesian models, specifically GPRs, to model the conditional distribution of each feature *f* given all other features. For each feature, we train a separate GPR model $g_{f}$ using the source domain inputs distributed across the participating clients.

We emphasize that these GPR models are not used for final prediction. Instead, they serve as intermediate models to compute confidence-based feature weights, which are later used in the federated training of the final adaptive weighted elastic net model.

The training data for a given feature *f* includes $X_{\neg f}^{S}$, the entire source domain data with the column corresponding to feature *f* removed, as inputs, and $X_{f}^{S}$, the corresponding feature vector, as labels. For new data points $X_{\neg f}^{T}=[x_{1,\neg f}^{t},\ldots ,x_{n_{t},\neg f}^{t}]$, the target domain data with the column for feature *f* removed, the goal is to predict the corresponding feature vector $X_{f}^{t}=[x_{1,f}^{t},\ldots ,x_{n_{t},f}^{t}]$.

For a specific source domain *i*, the vector containing feature *f* is denoted $X_{f}^{s_{i}}=[x_{1,f}^{s_{i}},\ldots ,x_{n_{i},f}^{s_{i}}]$, while the $\left(n_{i}\times (P-1)\right)$-matrix of remaining features is $X_{\neg f}^{s_{i}}=[x_{1,\neg f}^{s_{i}},\ldots ,x_{n_{i},\neg f}^{s_{i}}]$. Thus, for a given feature *f*, the GPR model $g_{f}$ provides a closed-form predictive distribution:


(1) $$g_{f}(X_{\neg f}^{T})∼N(K_{*}K^{-1}X_{f}^{S},K_{**}-K_{*}K^{-1}K_{*}^{⊤})$$

where


(2) $$K=k(X_{\neg f}^{S},X_{\neg f}^{S})+\sigma_{\epsilon }^{2}1_{n_{S}}K_{*}=k(X_{\neg f}^{S},X_{\neg f}^{T})$$

$$K_{**}=k(X_{\neg f}^{T},X_{\neg f}^{T})$$

Here, k(.,.) computes the linear kernel with the variance of the prior on the coefficients $\sigma_{k}^{2}$ between the given input matrices (Williams and Rasmussen 2006). Additionally, $n_{S}$ denotes the total number of samples in the entire source domain dataset $X^{S}$. Unlike traditional supervised regression models that predict a single value for a given input, GPRs provide a full predictive distribution as output (Seeger 2004), which we later use to compute feature weights. This GPR model involves two hyper-parameters that must be optimized for the best performance: the variance of the kernel, $\sigma_{k}^{2}$, and the variance of the additive Gaussian noise, $\sigma_{\epsilon }^{2}$, from the closed-form solution in Eq. (1). The optimal values of these hyper-parameters are determined by maximizing the marginal likelihood for each feature. For a specific source client *i*, and the covariance matrix $K=k(X_{\neg f}^{s_{i}},X_{\neg f}^{s_{i}})+\sigma_{\epsilon }^{2}1_{n_{i}}$ from Eq. (2), source client *i* maximizes:


(3) $$\text{log}\;L(X_{f}^{s_{i}}|X_{\neg f}^{s_{i}})=-\frac{1}{2}{\left(X_{f}^{s_{i}}\right)}^{⊤}K^{-1}X_{f}^{s_{i}}-\frac{1}{2}\text{log}\;|K|-\frac{n_{i}}{2}\text{log}(2\pi )$$

Training feature models is straightforward when both target and source domains are accessible simultaneously. However, significant challenges arise when these datasets are distributed.

The first challenge is that, if the source domain is distributed across multiple entities, the optimization of hyper-parameters shown in Eq. (3) cannot be performed across the entire source domain. The second, and more complex, challenge is that due to the distribution of the source and target domains, the closed-form solution of the GPR model (as shown in Eq. (1)) cannot be computed directly. Since GPRs are non-parametric ML algorithms, obtaining predictions requires computing the three matrices in Eq. (2), namely *K*, $K_{*}$, and $K_{**}$.

In our setting, computing $K_{**}$ is straightforward and can be performed locally by the owner of the target domain, as it requires only the target domain data. However, computing *K* and $K_{*}$ as well as the predictive mean of the feature model $K_{*}K^{-1}X_{f}^{S}$ presents significant challenges. Computing *K* is challenging because, although it only requires source domain data, the data are distributed across multiple entities, and the entire matrix product of *K* cannot be computed trivially. Instead, it must be computed collaboratively among all source domain owners while preserving privacy. Similarly, computing $K_{*}$ is challenging because it requires access to both source domain data and target domain data. Lastly, computing $K_{*}K^{-1}X_{f}^{S}$ is not straightforward because the feature column for the entire source domain data, $X_{f}^{S}$, is distributed between *N* source clients. Moreover, explicitly sharing $X_{f}^{S}$ would compromise privacy since the aggregator could reconstruct the data of all source clients after the feature model training phase is completed, as each feature is modeled independently.

FREDA addresses all of these challenges by employing secure aggregation and a customized masking scheme for matrix product computation (Hannemann *et al.* 2023).

#### 3.1.1 Federated hyper-parameter optimization

To optimize the GPR model hyper-parameters $\sigma_{k}^{2}$ (prior variance) and $\sigma_{\epsilon }^{2}$ (noise variance) in a federated setting, FREDA employs secure aggregation via zero-sum masking (Bonawitz *et al.* 2016). This method ensures privacy by having each client mask its data with random values that cancel out when aggregated, revealing only the global sum without exposing individual contributions. Each source client locally optimizes $\sigma_{k}^{2}$ and $\sigma_{\epsilon }^{2}$ by maximizing the marginal likelihood over its dataset. These values are then securely aggregated to compute global averages, effectively approximating joint optimization over the entire source domain.

#### 3.1.2 Federated GPR computation

In the GPR model prediction process, the most challenging components to compute are *K*, $K_{*}$, and their product with the global feature vector $X_{f}^{S}$, as these require access to both the source domain data and the target domain data to calculate the matrix product. A naive plaintext approach would compromise privacy. To overcome this, we utilize a framework for secure and private matrix product computation (Hannemann *et al.* 2023), which allows us to calculate the product of matrices from the source and target domains without disclosing their plaintext values.

***Privacy-preserving masking process***. We use special masking matrices to hide the input matrices of the matrix product and reveal only the result of this multiplication to the aggregator. This protects the privacy of the input matrices as well as their dimensionality. Specifically, both the source clients and the target client share a common seed to generate a shared mask matrix $M\in R^{d\times P}$, where *d* is a higher-dimensional space than the original feature space. To ensure that a left inverse exists, *M* is required to be full column rank. Each client *p* then locally computes a left inverse $L^{p}\in R^{P\times d}$ such that $L^{p}M=I_{P}$, and applies the mask to its data:


(4) $${\tilde{X}}^{p}=X^{p}L^{p}{\left(MM^{⊤}\right)}^{1/2},$$

where $X^{p}$ represents the local dataset of client *p* ($X^{p}=X^{s_{i}}$ for a source client or $X^{p}=X^{T}$ for the target client). The masked data ${\tilde{X}}^{p}$ is then sent to the aggregator.

***Secure gram matrix computation***. The aggregator computes the Gram matrix for all clients using the masked data. For a pair of clients *p* and *q*, the Gram matrix is computed as:


(5) $$\begin{array}{c} {\tilde{X}}^{p}{\left({\tilde{X}}^{q}\right)}^{⊤}=(X^{p}L^{p}{\left(MM^{⊤}\right)}^{1/2}){\left(X^{q}L^{q}{\left(MM^{⊤}\right)}^{1/2}\right)}^{⊤}\\ =X^{p}L^{p}{\left(MM^{⊤}\right)}^{1/2}{\left({\left(MM^{⊤}\right)}^{1/2}\right)}^{⊤}{\left(L^{q}\right)}^{⊤}{\left(X^{q}\right)}^{⊤}\\ =X^{p}(L^{p}M)(M^{⊤}{\left(L^{q}\right)}^{⊤}){\left(X^{q}\right)}^{⊤}\\ =X^{p}X^{q⊤} \end{array}$$

where the masking terms cancel out, revealing only the product ${\tilde{X}}^{p}{\left({\tilde{X}}^{q}\right)}^{⊤}$ without exposing the individual data of the clients.

Using the computed Gram matrix $G^{pq}$, the aggregator calculates *K* and $K_{*}$ as follows:


(6) $$\begin{array}{c} K=\sigma_{k}^{2}G^{pq}+\sigma_{\epsilon }^{2}1_{n_{S}}\\ K_{*}=\sigma_{k}^{2}G^{pq} \end{array}$$

***Computing the predicted mean***. Computing the predicted mean $K_{*}K^{-1}X_{f}^{S}$ is challenging as the aggregator does not have access to the label vector $X_{f}^{S}$ (feature column for the modeled feature $g_{f}$), which remains distributed across source clients. Explicitly sharing $X_{f}^{S}$ would also compromise privacy since the aggregator could reconstruct the data of all source clients after the feature model training phase is completed, as each feature is modeled independently. To address this, FREDA performs the following:

1. **Aggregator step**: The aggregator computes the intermediate matrix product $K_{*}K^{-1}$, then applies a random mask matrix $C\in R^{n_{t}\times n_{t}}$, resulting in the masked matrix:
  (7) $$\tilde{B}=CK_{*}K^{-1}$$

The masked matrix $\tilde{B}$ is split row-wise into sub-matrices corresponding to each source client’s data, denoted ${\tilde{B}}^{s_{i}}$. The aggregator sends ${\tilde{B}}^{s_{i}}$ to source client *i*, and separately sends $C^{-1}$ to the target client.

**Source client computation**: Each source client *i* receives their masked sub-matrix ${\tilde{M}}^{s_{i}}$ and locally computes:
  (8) $$v^{s_{i}}={\tilde{B}}^{s_{i}}X_{f}^{s_{i}},$$

where $X_{f}^{s_{i}}$ is the vector of values for feature *f* held by client *i*. The result $v^{s_{i}}\in R^{n_{t}\times 1}$ is then sent to the target client.

**Target client aggregation**: The target client aggregates the received vectors:
  (9) $$v=\sum_{i=1}^{N}v^{s_{i}},$$

and removes the mask using the inverse matrix $C^{-1}$:


(10) $$K_{*}K^{-1}X_{f}^{S}=C^{-1}v=C^{-1}\sum_{i=1}^{N}{\tilde{B}}^{s_{i}}X_{f}^{s_{i}}$$

This protocol enables the target client to compute the predicted mean without accessing any raw data from the source clients, thereby preserving data privacy. Additionally, the random masking step ensures that source clients cannot infer information about other clients or the full matrix $K_{*}K^{-1}$ from their sub-matrices.

***Computing the predicted variance***. To complete the predictive distribution, the predicted variance term $K_{**}-K_{*}K^{-1}K_{*}^{⊤}$ is computed by the aggregator using the Gram matrix $G^{pq}$ and sent to the target client. With both the predicted mean and variance available, the target client can reconstruct the full closed-form predictive distribution for feature *f* as given in Eq. (1).

### 3.2 Feature weight computation

For a sample from the target domain, denoted as $x_{m}^{t}$, and a given feature *f*, let $x_{m,f}^{t}$ represent the value of feature *f* in $x_{m}^{t}$, and $x_{m,\neg f}^{t}$ represent the values of all other features in $x_{m}^{t}$. Given $x_{m,\neg f}^{t}$, the feature model $g_{f}$ outputs a posterior distribution that describes the expected value of $x_{m,f}^{t}$ according to the dependency structure learned from the source domain. For a GPR model, this posterior is a normal distribution, which is directly obtained from the closed-form solution shown in Eq. (1) and collaboratively computed in the previous phase.

To evaluate how well the observed value $x_{m,f}^{t}$ fits the predicted distribution, we apply the confidence measure proposed by Jalali and Pfeifer (2016):


(11) $$c_{f}(x_{m}^{t})=2\Phi \left(\frac{\left|x_{m,f}^{t}-\mu_{g_{f}}(x_{m,\neg f}^{t})\right|}{\sigma_{g_{f}}(x_{m,\neg f}^{t})}\right)$$

where Φ denotes the cumulative distribution function of a standard normal distribution. Here, $\mu_{g_{f}}(x_{m,\neg f}^{t})$ and $\sigma_{g_{f}}(x_{m,\neg f}^{t})$ denote the mean and standard deviation of the predictive distribution computed in Eq. (1) for the input $x_{m,\neg f}^{t}$. Specifically, they correspond to the closed-form expressions $K_{*}K^{-1}X_{f}^{S}$ and $K_{**}-K_{*}K^{-1}K_{*}^{⊤}$, respectively, obtained from the feature model $g_{f}$. This confidence score represents the probability that a value as extreme as $x_{m,f}^{t}$, or more, relative to the predicted mean $\mu_{g_{f}}(x_{m,\neg f}^{t})$, occurs within the posterior distribution predicted by $g_{f}$.

The overall confidence for feature *f* in the target domain is then defined as the average of $c_{f}(x_{m}^{t})$ across all target domain samples:


(12) $$c_{f}=\frac{1}{n_{t}}\sum_{i=1}^{n_{t}}c_{f}(x_{m}^{t})$$

where $n_{t}$ is the total number of samples in the target domain. For each feature, $c_{f}$ quantifies how well the source-domain dependencies for feature *f* align with those in the target domain. Once the confidence scores for all features have been computed, the target client then computes the weight of feature *f* as follows:


(13) $$w_{f}={\left(1-c_{f}\right)}^{k}$$

Here, *k* is a hyper-parameter specified by the target client, with k>0. This hyper-parameter determines how the confidence scores are transformed into feature weights. As *k* increases, progressively higher penalties are applied to features with low confidence, while features with higher confidence are penalized less severely. Both the confidence score formulation and the transformation of confidence scores into feature weights are identical to those used in WENDA (Handl *et al.* 2019), which also relies on feature-wise predictive distributions to guide adaptation.

In our experiments, we empirically evaluate the performance of our framework with respect to the weighting parameter *k* and adjust its value accordingly (Section 4.4.3).

### 3.3 Federated weighted elastic net training

The remaining phases of our framework involves collaboratively training weighted elastic nets in a federated manner, to preserve the privacy of individual source clients. By using a weighted elastic net, source clients scale the contribution of each feature in their local data to the regularization term based on the feature weights computed by the target client in the previous step. The weighted elastic net solves the following optimization problem:


(14) $$\hat{\beta }=\underset{\beta }{\text{argmin}}\left(\left\|y-X\beta {\|}^{2}+\lambda J(\beta \right)\right)$$

where $\|y-X\beta {\|}^{2}$ represents the residual sum of squares on the source domain data, λ is the regularization parameter, and J(β) is the regularization term defined as:


(15) $$J(\beta )=\alpha \sum_{f=1}^{F}w_{f}|\beta_{f}|+\frac{1}{2}(1-\alpha )\sum_{f=1}^{F}w_{f}\beta_{f}^{2}$$

Training a weighted elastic net is conceptually similar to training a neural network with a single linear layer in a federated setting, where the coefficient for each feature is scaled by its corresponding confidence-based weight. Since all source clients share the same fixed feature weights, the federated training focuses on updating the model coefficients collaboratively.

We employ the FedAvg algorithm (McMahan *et al.* 2017) to train the weighted elastic net in a privacy-preserving manner. Specifically, each client performs local gradient-based updates using their own data, and the resulting model updates are securely aggregated at the central server (Tajabadi *et al.* 2024) using the secure aggregation protocol (Bonawitz *et al.* 2016). The aggregated global model is then broadcast back to all clients for the next training round. To reflect the differing amounts of data across clients, each client’s contribution to the global model is weighted proportionally to its local sample size. This setup ensures that clients with larger and more representative data pools have a stronger influence on the global model, which can be particularly beneficial in imbalanced scenarios.

As shown in Equations (14, 15), FREDA has two external parameters: the proportion of $L_{1}$ and $L_{2}$ penalties in the weighted elastic net α, and the regularization parameter λ. Following Handl *et al.* (2019), we fix α=0.8.

#### 3.3.1 Optimal lambda prediction

The regularization parameter λ plays a critical role in domain adaptation by balancing feature penalty strength and model learning (Handl *et al.* 2019). If λ is too small, the model may not sufficiently penalize feature differences, while an overly large λ could dominate the objective function, limiting meaningful adaptation. Since cross-validation is impractical in an unsupervised setting where target labels are unavailable, FREDA adopts the prior knowledge approach from Handl *et al.* (2019), leveraging domain similarities known by the target client.

The target client partitions its data into subsets $X^{t_{1}}$ and $X^{t_{2}}$. Source clients federatively train weighted elastic nets across a range of λ values, and the target client selects the best-performing model for $X^{t_{1}}$ based on MAE, assuming labels for this subset are available. A simple linear model is then fitted to capture the relationship between domain similarity and the optimal λ values obtained from source-trained models. This model is used to predict λ values for $X^{t_{2}}$, which are then sent back to source clients for the final training phase.

#### 3.3.2 Final adaptive model training

The source clients federatively train the final weighted elastic net models by selecting the regularization parameter λ based on the predicted values received from the target client in the previous step.

The model obtained at the end of this step is sent to the target client by the aggregator for the final prediction task on the target domain data.

## 4 Results

To evaluate the performance of our proposed method, we provide a benchmark on the problem of age prediction from DNA methylation data across multiple tissues. This section presents a detailed comparison of FREDA with existing baselines, highlighting its effectiveness in preserving data privacy while achieving competitive predictive accuracy.

### 4.1 Implementation

We implemented our framework in Python 3.8.18, the source code to reproduce the experiments is available on GitHub (https://github.com/mdppml/FREDA). For the feature models, we implemented our own GPR models and used the Python package GPy (https://github.com/SheffieldML/GPy, gpy 2012) to compute the optimal values for the hyper-parameter optimization explained in Section 3.1.1. As for the weighted elastic nets, we used TensorFlow 2.13.1 with custom kernel regularization. All federated processes, including source clients, target client, and aggregator, are simulated locally to enable reproducible evaluations. To encourage adoption in broader settings, we also provide a task-agnostic version of our framework, available at https://github.com/mdppml/FREDA-CV. This implementation removes the need for prior domain similarity knowledge by using cross-validation to select the regularization parameter λ.

### 4.2 Dataset and pre-processing

We used DNA methylation and donor age data from TCGA (Weinstein *et al.* 2013) and GEO (Edgar *et al.* 2002), following the exact preprocessing steps of Handl *et al.* (2019). This included imputing missing values (<0.5% of samples) and reducing dimensionality from 466 094 to 12 980 features. Ages were transformed using Horvath’s method (Horvath 2013) to account for nonlinear methylation changes, then standardized. The dataset was split into a source set (1866 samples from 19 tissues, ages 0–103) and a target set (1001 samples from 13 tissues, including unseen ones like cerebellum). As in Handl *et al.* (2019), similar tissue types were aggregated to ensure sufficient sample sizes. Detailed preprocessing steps is available in the Supplementary Material, available as supplementary data at *Bioinformatics* online.

### 4.3 Baselines

We compared FREDA with two baselines: the state-of-the-art unsupervised domain adaptation method WENDA (Handl *et al.* 2019), and the non-adaptive model (Horvath 2013).

#### 4.3.1 WENDA baseline

WENDA (weighted elastic net for domain adaptation) is a state-of-the-art method for unsupervised domain adaptation on small-scale, high-dimensional biological datasets (Handl *et al.* 2019). Like our approach, it leverages the dependency structure between features across source and target domains, penalizing discrepant features while emphasizing robust ones. Despite its strong performance over non-adaptive models, WENDA assumes simultaneous access to both domains, hence it is only suitable for non-private settings.

WENDA has three key parameters: the weighting parameter *k*, the elastic net mixing parameter α, and the regularization parameter λ. Following Handl *et al.* (2019), we fix α=0.8, while λ is computed using prior knowledge on tissue similarity (WENDA-pn), as cross-validation on the target domain is infeasible in an unsupervised setting. For *k*, we select k=3 based on both our experiments and prior work (Handl *et al.* 2019).

#### 4.3.2 Non-adaptive baseline

For our non-adaptive baseline, we adopt the method proposed by Horvath (2013), which combines the elastic net with a least-squares fit. The idea is to first fit a standard elastic net and then apply a linear least-squares fit based only on features that obtained non-zero coefficients in the elastic net. This baseline was first proposed by Horvath (2013) for age prediction from DNA methylation data, where he demonstrated that using an elastic net followed by a least-squares fit resulted in improved performance on his dataset. We refer to this non-adaptive method as *en-ls*.

### 4.4 Setup for FREDA

We consider a distributed setting with multiple source domain clients, a target client, and an aggregator, which has no data. The labeled source domain data is distributed across 2, 4, or 8 source clients, and we evaluate FREDA in each of these settings.

#### 4.4.1 Data distribution

Source domain data are assigned uniformly at random among the source clients. Given that the DNA methylation dataset contains 1866 training samples, each client receives approximately 933, 466, or 233 samples in the 2-, 4-, and 8-client settings, respectively.

To assess the robustness of FREDA, we do not consider tissue types when distributing data. Due to the inherent imbalance of DNA methylation data across tissues, this results in some clients having only a few or no samples from certain tissues.

#### 4.4.2 Setup for weighted elastic net models

The weighted elastic net model is trained for 100 global iterations, with source clients updating their local models for 20 epochs per iteration before the global model is securely updated. To improve convergence, we apply an exponential learning rate decay across iterations, starting at $1\times 10^{-4}$ and decreasing to $1\times 10^{-5}$ by the final iteration (Yan *et al.* 2022).

#### 4.4.3 Parameter selection in FREDA

FREDA has three key parameters: the weighting parameter *k*, the elastic net mixing parameter α, and the regularization parameter λ. We fix α=0.8 as a design choice and determine λ using the prior knowledge approach (Section 3.3.1) proposed by Handl *et al.* (2019).

For tissue similarity calculations, we use data from the GTEx consortium, which provides genotype and gene expression data across 42 human tissues (GTEx Consortium 2017), following the methodology in Handl *et al.* (2019). In the federated setting, only the target domain owner requires access to tissue similarity information.

To evaluate performance, we follow the evaluation strategy of Handl *et al.* (2019) for WENDA-pn, iteratively splitting test tissues into subsets: one for fitting domain similarity relationships and another for evaluation (Section 3.3.1). We iterate over all three-tissue combinations with at least 20 training samples, assessing performance on the remaining tissues. Based on our experiments, the optimal weighting parameter is k=3.

### 4.5 Experiments

We compare the performance of FREDA against WENDA-pn and the non-private, non-adaptive baseline model *en-ls*, as described in Section 4.3. The main performance metric is the mean absolute error (MAE) of the predicted chronological ages of the tissues. For WENDA-pn, we calculate the MAE only on samples not used for fitting the tissue similarity–λ relationship, reporting the mean and standard deviation across all splits. Similarly, for FREDA, we report the MAE exclusively for the target client’s tissues that were not part of the similarity–λ fit, along with the mean and standard deviation over all splits.

For the non-adaptive baseline *en-ls*, Handl *et al.* emphasize that the heterogeneous nature of the data and the random splitting of the training data used for 10-fold cross-validation significantly influence its performance. Therefore, we follow their approach and report the mean ± standard deviation over 10 runs for *en-ls*. For WENDA-pn, the mean ± standard deviation is calculated over all splits of the test tissues where the tissue of interest was included in the evaluation set. For FREDA, we report the mean ± standard deviation for each setting (2, 4, and 8 sources) over five different uniform random distributions of source data across the source parties, considering all splits where the tissue of interest was included in the evaluation set.

For WENDA-pn, Handl *et al.* (2019) treat each tissue in the test dataset as a separate target domain, training the final weighted elastic net models independently for each tissue. Specifically, Handl *et al.* (2019) compute the average confidences, as defined in Equation (12), only over the samples of the same tissue and train a separate model for each tissue, always using the entirety of the training (source) data but applying tissue-specific feature weights. We follow the same approach for FREDA in all our experiments, where the clients inside the federated learning system train a separate weighted elastic net model for each tissue in the target domain (for further information see Section 3).

**Figure 2.**
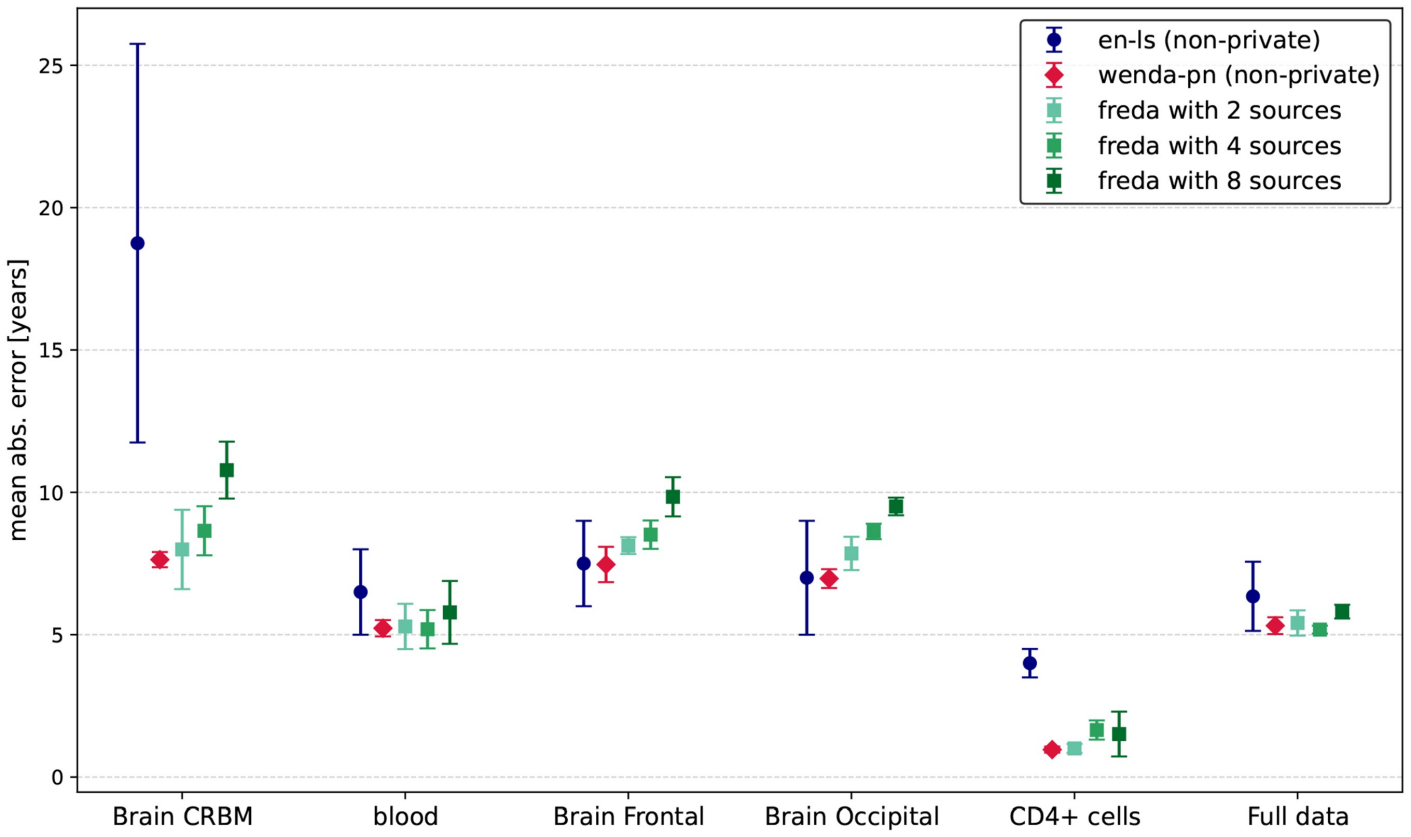
MAE per target tissue and on the full target dataset for *en-ls*, WENDA-pn (k=3), and FREDA (k=3) with 2, 4, and 8 source parties.

The performance of *en-ls*, WENDA-pn, and FREDA for 2, 4, and 8 source parties on the relevant tissues of the target domain, as well as on all samples of the target domain data, is shown in Fig. 2. For the full target dataset, the non-private baseline methods *en-ls* and WENDA-pn yield an MAE of 6.34±1.21 and 5.31±0.29, respectively. These results indicate that when the entire target domain data is considered, WENDA-pn provides only a slight improvement in performance compared to the non-adaptive *en-ls*. The effect of distribution shift is most visible when we observe the performance of our baselines on cerebellum samples. As shown in Fig. 2, the non-adaptive *en-ls* yields a significantly higher MAE on cerebellum samples compared to other tissues.

Figure 4 shows the predicted versus true ages for the samples of the target domain data, colored by tissue, for FREDA with k=3 for 2, 4, and 8 source parties, *en-ls* and WENDA-pn, respectively. From Fig. 4d and e, we can clearly see that both non-private methods perform well on most tissues, except for *en-ls* on cerebellum samples. As shown in Fig. 4d, the ages predicted by *en-ls* for cerebellum samples are consistently lower than the true chronological ages. In contrast, Fig. 4e demonstrates that WENDA-pn achieves much closer alignment between the predicted and true ages for cerebellum samples.

Additionally, for the remaining target domain tissues, the predictions of WENDA-pn are comparable to those of *en-ls*, as confirmed by the quantitative results in Fig. 2. WENDA-pn not only yields significantly lower errors than *en-ls* on cerebellum samples but also maintains similar or better performance on other test tissues. Specifically, on cerebellum samples, *en-ls* produces a MAE of 7.63±0.26. These results highlight the significance of improving prediction performance on cerebellum samples without a drop in performance on other tissues.

Our experimental results, present in Figs 2 and 4, are consistent with the findings of Handl *et al.* (2019), who highlight the difficulty of predicting the age of cerebellum samples. These samples are not represented in the training data and are known to be biologically distinct, even from other brain tissues, in terms of function and gene expression patterns (Fraser *et al.* 2005; GTEx Consortium 2017). Hence, our evaluation focuses on whether federated privacy-preserving domain adaptation, as implemented by FREDA, can achieve comparable performance on these samples to the non-private method WENDA-pn.

For the full target dataset, FREDA achieves a MAE of 5.41±0.44, 5.41±0.44, and 5.81±0.24 for the 2, 4, and 8 source domain settings, respectively. These results indicate that, when considering the full target domain data, FREDA provides a performance level almost identical to that of WENDA-pn and consistently better than *en-ls* across all configurations, despite operating in a distributed environment.

### 4.6 Effect of data distribution on performance

To evaluate the robustness of FREDA under more realistic deployment scenarios, we extended our benchmark by introducing non-uniform data distributions across source clients. While our primary experiments used a uniform random distribution of samples among clients, real-world federated settings often exhibit substantial data imbalance. In our context, where the task is regression and the data is already highly tissue-imbalanced, we focus on *sample-wise imbalance*.

We simulate two increasingly imbalanced scenarios in the four source-client setting. In the first, mildly imbalanced setting, clients receive data according to a skewed distribution of [0.5, 0.2, 0.2, 0.1], and in the second, highly imbalanced setting, sample proportions follow [0.533, 0.266, 0.133, 0.068], where each client has roughly double the number of samples of the next. These distributions mimic real-world scenarios where certain institutions contribute significantly more data than others.

**Figure 3.**
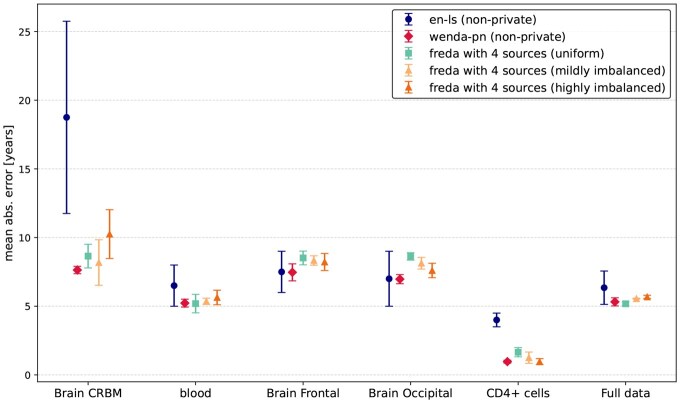
MAE per target tissue, as well as on full target data, for *en-ls*, WENDA-pn (k=3), and FREDA (k=3) under uniform and two imbalanced four-client data distributions.

As shown in Fig. 3, FREDA maintains strong predictive performance even under considerable sample imbalance. Although minor degradations in MAE can be observed specifically for cerebellum samples, the overall performance remains competitive with non-private baselines.

**Figure 4.**
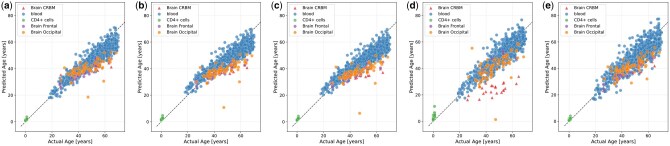
Predicted versus true chronological age under various settings. Figures (a), (b), and (c) correspond to FREDA with k=3 for 2, 4, and 8 source parties, respectively. Predictions are averaged over all splits where the tissue of interest was included in the evaluation set, as well as over five different distributions for each setting. Panels (d) and (e) correspond to *en-ls* and WENDA-pn, respectively. For *en-ls*, predictions are averaged over 10 runs of 10-fold cross-validation, while for WENDA-pn, predictions are averaged over all splits where the tissue of interest was included in the evaluation set.

## 5 Discussion

Cerebellum samples continue to represent the most challenging case for age prediction under domain shift, consistent with prior findings (Fraser *et al.* 2005, GTEx Consortium 2017, Handl *et al.* 2019). These samples differ biologically from other brain and non-brain tissues, and are not well represented in training data. Despite this, FREDA achieves comparable performance to the non-private method WENDA-pn in the 2- and 4-source scenarios, with MAEs of 7.99±1.39 and 8.64±0.86, respectively. In contrast, the non-adaptive baseline *en-ls* consistently underestimates the ages of cerebellum samples, leading to poor performance on this difficult target domain.

Across all test tissues, FREDA closely matches the performance of WENDA-pn while significantly outperforming *en-ls*. Notably, despite being trained in a privacy-preserving federated setting, FREDA maintains high predictive performance and effectively captures domain-specific distribution shifts. This confirms that the federated adaptation strategy does not sacrifice performance, even though clients operate under strict data privacy constraints.

We also investigated the impact of scaling to more source clients. As the number of source domains increases from 2 to 8, a slight degradation in performance is observed on cerebellum samples, with MAE increasing to 10.77±0.99. This suggests that partitioning the source data too finely can hinder adaptation performance, possibly due to reduced statistical power per client. Nonetheless, even in the 8-party case, FREDA still outperforms the non-private and non-adaptive baseline.

To further assess robustness in more realistic settings, we extended our evaluation with experiments using imbalanced data distributions across source clients. These scenarios simulate common federated learning situations where data contributions vary widely across institutions. Interestingly, we observe that predictive performance on some target tissues actually improves as the degree of imbalance increases. We attribute this to two main factors: first, our randomized encoding scheme for feature modeling allows the aggregator to compute global feature statistics as if all data were pooled, despite privacy constraints. Second, during the federated training of the weighted elastic nets, weighted aggregation implicitly favors clients with larger datasets, which can lead to more stable model updates when the dominant client has a representative sample distribution. Performance on cerebellum, however, remains more sensitive to imbalance possibly due to its distinct biological characteristics. Despite this, FREDA maintains competitive performance, demonstrating robustness to imbalanced real-world settings.

Together, these findings demonstrate that FREDA successfully balances privacy, performance, and adaptability, even in challenging domain adaptation tasks and realistic federated learning settings.

## 6 Conclusion

In this article, we introduced FREDA, the first privacy-preserving framework for federated unsupervised domain adaptation in regression tasks on high-dimensional, small-scale biological datasets. FREDA enables multiple entities to collaboratively model complex feature relationships while maintaining complete data privacy. By combining randomized encoding and secure aggregation, it addresses the challenge of training Gaussian processes in distributed settings, eliminating the need for pooled pairwise computations on non-shareable data.

Our evaluation on an age prediction task from DNA methylation data demonstrates that FREDA achieves performance comparable to non-private methods, including on challenging tissues such as cerebellum, while preserving data privacy. In addition, we observe that FREDA remains robust under increasingly imbalanced data distributions.

While FREDA demonstrates competitive performance to the non-private state-of-the-art even in distributed settings, we acknowledge that training a separate feature model for each feature in high-dimensional settings can be computationally intensive. However, there are several directions to improve scalability that we plan to explore in future work. First, since feature models are independent, they can be trained in parallel across multiple processors or compute nodes to reduce runtime. Second, recent work (Hannemann *et al.* 2025) proposes a more efficient masking strategy for the same randomized encoding framework used in FREDA, which reduces computation time significantly by speeding up the masking process.

## Supplementary Material

btaf465_Supplementary_Data

## References

[btaf465-B1] Angermueller C , Pärnamaa T, Parts L et al Deep learning for computational biology. Mol Syst Biol 2016;12:878. 27474269 10.15252/msb.20156651 PMC4965871

[btaf465-B2] Bonawitz K , Ivanov V, Kreuter B et al Practical secure aggregation for federated learning on user-held data. arXiv, arXiv:1611.04482, 2016, preprint: not peer reviewed.

[btaf465-B3] Edgar R , Domrachev M, Lash AE. Gene expression omnibus: NCBI gene expression and hybridization array data repository. Nucleic Acids Res 2002;30:207–10. 11752295 10.1093/nar/30.1.207 PMC99122

[btaf465-B4] Farahani A , Voghoei S, Rasheed K et al A brief review of domain adaptation. In: Stahlbock R, Weiss GM, Abou-Nasr M et al. (eds), Advances in Data Science and Information Engineering. Transactions on Computational Science and Computational Intelligence. Cham: Springer. 10.1007/978-3-030-71704-9_65

[btaf465-B5] Feng H , You Z, Chen M et al Kd3a: unsupervised multi-source decentralized domain adaptation via knowledge distillation. In: Meila M, Zhang T (eds.), *Proceedings of the 38th International Conference on Machine Learning*. Vol. 139 of *Proceedings of Machine Learning Research, 18–24 July 2021, Virtual Event (2021)*. PMLR, 2021, 3274–83.

[btaf465-B6] Fraser HB , Khaitovich P, Plotkin JB et al Aging and gene expression in the primate brain. PLoS Biol 2005;3:e274. 16048372 10.1371/journal.pbio.0030274 PMC1181540

[btaf465-B7] Ganin Y , Lempitsky V. Unsupervised domain adaptation by backpropagation. In: Bach F, Blei D (eds), Proceedings of the 32nd International Conference on Machine Learning. Lille, France: PMLR, 1180–9.

[btaf465-B8] Ganin Y , Ustinova E, Ajakan H et al Domain-adversarial training of neural networks. J Mach Learn Res 2016;17:1–35.

[btaf465-B9] gpy. GPY: A Gaussian Process Framework in Python. Sheffield Machine Learning Group. 2012.

[btaf465-B10] Greener JG , Kandathil SM, Moffat L et al A guide to machine learning for biologists. Nat Rev Mol Cell Biol 2022;23:40–55. 34518686 10.1038/s41580-021-00407-0

[btaf465-B11] GTEx Consortium. Genetic effects on gene expression across human tissues. Nature 2017;550:204–13. 29022597 10.1038/nature24277 PMC5776756

[btaf465-B12] Handl L , Jalali A, Scherer M et al Weighted elastic net for unsupervised domain adaptation with application to age prediction from DNA methylation data. Bioinformatics 2019;35:i154–63. 31510704 10.1093/bioinformatics/btz338 PMC6612879

[btaf465-B13] Hannemann A , Ünal AB, Swaminathan A et al A privacy-preserving framework for collaborative machine learning with kernel methods. In: 2023 5th IEEE International Conference on Trust, Privacy and Security in Intelligent Systems and Applications (TPS-ISA), Atlanta, GA, USA, 2023, 82–90

[btaf465-B14] Hannemann A , Swaminathan A, Ünal AB et al Private, efficient and scalable kernel learning for medical image analysis. In: International Meeting on Computational Intelligence Methods for Bioinformatics and Biostatistics. Springer, 2025, 81–95.

[btaf465-B15] Horvath S. DNA methylation age of human tissues and cell types. Genome Biol 2013;14:R115–20. 24138928 10.1186/gb-2013-14-10-r115 PMC4015143

[btaf465-B16] Jalali A , Pfeifer N. Interpretable per case weighted ensemble method for cancer associations. BMC Genomics 2016;17:501–10. 27435615 10.1186/s12864-016-2647-9 PMC4952276

[btaf465-B17] Jiang E , Zhang YJ, Koyejo S. Principled federated domain adaptation: gradient projection and auto-weighting. In: The Twelfth International Conference on Learning Representations (ICLR 2024); 2024 Apr 30–May 3; Vienna, Austria, 2024.

[btaf465-B18] Li MM , Huang K, Zitnik M. Graph representation learning in biomedicine and healthcare. Nat Biomed Eng 2022;6:1353–69. 36316368 10.1038/s41551-022-00942-x PMC10699434

[btaf465-B19] Liang J , Hu D, Wang Y et al Source data-absent unsupervised domain adaptation through hypothesis transfer and labeling transfer. IEEE Trans Pattern Anal Mach Intell 2022;44:8602–17. 34383644 10.1109/TPAMI.2021.3103390

[btaf465-B20] Liu X , Chen Z, Zhou L et al UFDA: universal federated domain adaptation with practical assumptions. In: Proceedings of the 38th Annual AAAI Conference on Artificial Intelligence (AAAI-24); 2024 Feb 20–27; Vancouver, Canada. Vol. 38, 2024, 14026–34.

[btaf465-B21] Long M , Cao Y, Wang J et al Learning transferable features with deep adaptation networks. In: Bach F, Blei D (eds), Proceedings of the 32nd International Conference on Machine Learning. Lille, France: PMLR, 97–105.

[btaf465-B22] Long M , Zhu H, Wang J et al Unsupervised domain adaptation with residual transfer networks. In: Proceedings of the 30th International Conference on Neural Information Processing Systems. Barcelona, Spain: Curran Associates Inc., 136–144.

[btaf465-B23] Long M , Cao Z, Wang J et al Conditional adversarial domain adaptation. In: Proceedings of the 32nd International Conference on Neural Information Processing Systems. Montréal, Canada: Curran Associates Inc., 1647–57.

[btaf465-B24] Lundberg SM , Nair B, Vavilala MS et al Explainable machine-learning predictions for the prevention of hypoxaemia during surgery. Nat Biomed Eng 2018;2:749–60. 31001455 10.1038/s41551-018-0304-0 PMC6467492

[btaf465-B25] McMahan B , Moore E, Ramage D et al Communication-efficient learning of deep networks from decentralized data. In: *Proceedings of the 20th International Conference on Artificial Intelligence and Statistics*, Fort Lauderdale, FL, USA, 20-22 April 2017, 2017, 1273–82.

[btaf465-B26] Orouji S , Liu MC, Korem T et al Domain adaptation in small-scale and heterogeneous biological datasets. Sci Adv 2024;10:eadp6040. 10.1126/sciadv.adp6040 39705361 PMC11661433

[btaf465-B27] Peng X , Huang Z, Zhu Y et al Federated adversarial domain adaptation. In: International Conference on Learning Representations, Virtual Event, 2020.

[btaf465-B28] Poplin R , Varadarajan AV, Blumer K et al Prediction of cardiovascular risk factors from retinal fundus photographs via deep learning. Nat Biomed Eng 2018;2:158–64. 31015713 10.1038/s41551-018-0195-0

[btaf465-B29] Seeger M. Gaussian processes for machine learning. Int J Neural Syst 2004;14:69–106. 15112367 10.1142/S0129065704001899

[btaf465-B30] Sener O , Song HO, Saxena A et al Learning transferrable representations for unsupervised domain adaptation. Adv Neural Inf Process Syst 2016;29.

[btaf465-B31] Sun B , Feng J, Saenko K. Return of frustratingly easy domain adaptation. In: *Proceedings of the AAAI Conference on Artificial Intelligence*, Vol. 30, Phoenix, Arizona, USA, 2016.

[btaf465-B32] Sun B , Huo H, Yang Y et al PartialFed: cross-domain personalized federated learning via partial initialization. Adv Neural Inf Process Syst 2021;34:23309–20.

[btaf465-B33] Tajabadi M , Martin R, Heider D. Privacy-preserving decentralized learning methods for biomedical applications. Comput Struct Biotechnol J 2024;23:3281–7. 39296807 10.1016/j.csbj.2024.08.024 PMC11408144

[btaf465-B34] Thumuluri V , Almagro Armenteros JJ, Johansen A et al DeepLoc 2.0: multi-label subcellular localization prediction using protein language models. Nucleic Acids Res 2022;50:W228–34. 10.1093/nar/gkac278 35489069 PMC9252801

[btaf465-B35] Valentini G , Tagliaferri R, Masulli F. Computational intelligence and machine learning in bioinformatics. Artif Intell Med 2009;45:91–6. 10.1016/j.artmed.2008.08.014 18929473

[btaf465-B36] Wang M , Deng W. Deep visual domain adaptation: a survey. Neurocomputing (Amst) 2018;312:135–53.

[btaf465-B37] Weinstein JN , Collisson EA, Mills GB et al; Cancer Genome Atlas Research Network. The cancer genome atlas pan-cancer analysis project. Nat Genet 2013;45:1113–20. 24071849 10.1038/ng.2764 PMC3919969

[btaf465-B38] Weng Z , Yang X, Li A et al Open-VCLIP: transforming clip to an open-vocabulary video model via interpolated weight optimization. In: *Proceedings of the 40th International Conference on Machine Learning (ICML 2023); 2023 Jul 23–29; Honolulu, Hawaii, USA.* PMLR, 2023, 36978–89.

[btaf465-B39] Williams CK , Rasmussen CE. Gaussian Processes for Machine Learning, Vol. 2. Cambridge, MA: MIT Press, 2006.

[btaf465-B40] Wu J , He J, Wang S et al Distribution-informed neural networks for domain adaptation regression. Adv Neural Inf Process Syst 2022;35:10040–54.

[btaf465-B41] Yan G , Wang H, Li J. Seizing critical learning periods in federated learning. In: *Proceedings of the 36th AAAI Conference on Artificial Intelligence (AAAI-22), Virtual Event (2022).* Palo Alto, CA: AAAI Press, 2022, 8788–96.

[btaf465-B42] Yue Z , Sun Q, Zhang H. Make the U in UDA matter: invariant consistency learning for unsupervised domain adaptation. Adv Neural Inf Process Syst 2023;36:26991–7004.

