# Supplementary material for: Privacy-preserving federated unsupervised domain adaptation with application to age prediction from DNA methylation data
Source: Bioinformatics. 2025 Aug 22;41(10):btaf465. doi: 10.1093/bioinformatics/btaf465 (PMC12512134; doi:10.1093/bioinformatics/btaf465)
Supplement: btaf465_Supplementary_Data [file btaf465_supplementary_data.pdf]

Privacy-Preserving Federated Unsupervised Domain Adaptation for Regression on Small-Scale and High-Dimensional Biological Data

Cem Ata Baykara[1,*], Ali Burak Ünal[1,2], Nico Pfeifer[2], Mete Akgün[1,2]

[1]Medical Data Privacy and Privacy-Preserving Machine Learning, University of Tübingen, Tübingen, Germany

[2]Institute for Bioinformatics and Medical Informatics, University of Tübingen, Tübingen, Germany

[*]Corresponding author: cem.baykara@uni-tuebingen.de

Method Overview

To provide a overview of the FREDA framework, we include a high-level pseudo-code summary in Algorithm 1. This representation outlines the key computational phases of the method, including distributed feature modeling, confidence-based feature weighting, regularization parameter selection, and federated model training. The algorithm illustrates the interaction between the source clients, target client, and the aggregator.

Algorithm 1 FREDA: Federated Unsupervised Domain Adaptation

Require: Source clients $\{S_1, ..., S_N\}$ with $\{X^{s_i}, y^{s_i}\}$, target client T with X^T, weighting parameter k
Ensure: Final adaptive model coefficients β_{final} at the target client

Phase 1: Distributed Feature Modeling
for each feature $f = 1$ to $\mathcal{P}$ **do**
  for each source client S_i **do**
    Compute $(\sigma_k^2)^{S_i}$, $(\sigma_\epsilon^2)^{S_i}$ on $X^{s_i}_{\neg f}$
  end for
  Aggregator securely aggregates hyper-parameters to compute global σ_k^2, σ_ϵ^2
  Securely compute $g_f(X^T_{\neg f}) \sim \mathcal{N}(\mu_{g_f}, \sigma^2_{g_f})$
  Target client computes confidence score c_f and derives feature weight $w_f = (1 - c_f)^k$
end for
Target client sends all feature weights $\{w_1, ..., w_{\mathcal{P}}\}$ to source clients via aggregator
Phase 2: Optimal λ Selection
Target client partitions X^T into X^{t_1} and X^{t_2}
for each λ in a predefined set **do**
  Source clients train a weighted elastic net model β_λ in a federated manner using weights $\{w_f\}$
end for
Target client selects the best λ^* based on X^{t_1} and communicates it to source clients
Phase 3: Final Adaptive Model Training
Source clients train final model β_{final} using weights $\{w_f\}$ and λ^*
Aggregator sends β_{final} to the target client
Target client uses β_{final} for inference on X^T

Dataset and Pre-Processing

We utilized DNA methylation data and donor age information from two main sources: the Cancer Genome Atlas (TCGA) [9] and the Gene Expression Omnibus (GEO) [2]. For consistency and ease of comparison, we follow the exact preprocessing steps described by Handl et al. [4], including the imputation of missing values, which constituted less than 0.5% of all samples, as well as dimensionality reduction on the features, reducing the initial set of 466,094 features to 12,980.

We apply a transformation to the chronological ages based on the method proposed by Horvath [6]. For all ages in the training set, we used the function:

$$F(y) = \begin{cases} \log(y+1) - \log(y_{\text{adult}}+1), & \text{if } y \leq y_{\text{adult}} \\ (y - y_{\text{adult}})/(y_{\text{adult}}+1), & \text{otherwise} \end{cases}$$

where $y_{\text{adult}} = 20$ represents the adult age threshold prior to training. After training, we reversed this transformation using its inverse function, F^{-1}. This transformation is logarithmic for ages below y_{adult} and linear for ages above, reflecting that methylation patterns change more rapidly during childhood and adolescence than in adulthood. Finally, we standardized all data to have zero mean and unit variance.

The dataset was then divided into a training (source) set of 1,866 samples and a test (target) set of 1,001 samples. The training set included samples from 19 different tissues, predominantly blood, with donors' ages ranging from 0 to 103 years. The test set initially contained samples from 13 different tissues, including blood and tissues not represented in the training set, such as those from the cerebellum. Following the approach of Handl et al. [4], we aggregated similar tissue types, such as combining 'blood','whole blood', and 'menstrual blood', as well as 'Brain Medial Frontal Cortex' and 'Brain Frontal Cortex', to ensure sufficient sample sizes per tissue type.

Security Analysis

We consider the security guarantees of our framework based on the following assumptions:

1. The aggregator is semi-honest, meaning it follows the protocol but may attempt to infer sensitive information from observed data.
2. Participating data owners are semi-honest, meaning they follow the protocol steps but may try to infer sensitive information from observed data or intermediate results.
3. The communication channel between the aggregator and the clients is secure (either through TLS or SSL). It is not possible for other clients to perform man-in-the-middle attacks or eavesdrop on the exchanged messages.

The assumption of a semi-honest aggregator and semi-honest clients is widely regarded as a standard in privacy-preserving machine learning [8, 7, 3]. Based on these assumptions, we analyze the information which can be inferred by the participants during each phase of our framework.

Security of the Federated Feature Model Training

The training of feature models consists of two key steps: federated hyper-parameter optimization and federated GPR training.

Security of the Federated Hyper-parameter Optimization

This step is performed collaboratively between the source clients and the aggregator. The local data of the source clients are never exposed directly to either the aggregator or other data owners during this process. The only information exchanged during this step includes the optimal values of the prior variance on the coefficients σ_k^2 and the variance of the additive noise σ_ϵ^2. These values are computed locally by each source client for each feature.

The use of secure aggregation, implemented via zero-sum masking as formally proven in [1], ensures that the semi-honest aggregator cannot infer individual hyper-parameter values for any source client. Instead, the aggregator only gains access to the aggregated sum of hyper-parameters for each feature, which contains no exploitable information about the local datasets.

Security of the Federated GPR Training

This step is performed collaboratively between all participants, which include the target client, source clients, and the aggregator.

Aggregator's Perspective. The information disclosed to the aggregator is limited to the number of local data samples held by each source client, as indicated by the size of each sub-matrix. This information is necessary for the correct partitioning of the intermediate mean matrix K_*K^{-1}, but it does not reveal any details about the content of the local data samples. Due to the masking process employed, the aggregator cannot infer any information regarding the local datasets of the source or target clients. The only data accessible to the aggregator is the resulting Gram matrix G^{pq}, which is computed from the masked matrices. The security of this masking process, implemented within the FLAKE framework, is formally proven in [5].

Source Clients' Perspective. The information available to each source client is limited to the respective sub-matrix they receive from the aggregator, which is derived from K_*K^{-1}. The aggregator masks the intermediate matrix product by multiplying it with a mask matrix C, resulting in the masked matrix $\tilde{B} = CK_*K^{-1}$. This masked matrix is then split into sub-matrices and sent to the respective source clients. Since each source client only has access to their own sub-matrix, they cannot infer any information about the overall matrix or the data of other clients without having access to the masking matrix C.

Target Client's Perspective. The information available to the target client includes the masked mean vectors sent by the source clients which it aggregates as $v = \sum_{i=1}^{N} v^{s_i}$, the inverse of the masking matrix C^{-1}, and the predicted variance $K_{**} - K_*K^{-1}K_*^\top$ sent by the aggregator. To remove the mask on the mean vectors, the target client computes:

$$K_*K^{-1}X_f^S = C^{-1}v = C^{-1}\sum_{i=1}^{N}\tilde{B}^{s_i}X_f^{s_i}.$$

d Since the individual vectors from the source clients are aggregated during this process, the target client cannot infer any meaningful information about the local datasets of the source clients. Regarding the predicted variance $K_{**} - K_*K^{-1}K_*^\top$, although the target client has access to K_{**}, it can compute $-K_*K^{-1}K_*^\top$. However, without access to the individual data matrices of the source clients, the target client cannot infer any meaningful information, as there are infinitely many possible source data matrices that would result in the same K and K_* [5].

Security of the Feature Weight Computation

This step is performed locally by the target client. At the end of this process, the target client sends the computed feature weights for all tissues to the aggregator. The aggregator then distributes these weights to the source clients.

The local data of the target client is never directly exposed to either the aggregator or the source clients during this process. The only information shared with the aggregator and source clients consists of the feature weights for each tissue in the target domain. These feature weight vectors are aggregate values, computed by the target client by averaging the feature weights of the samples belonging to each tissue within the target domain data. Consequently, it is not possible for the aggregator or the source clients to infer any meaningful information on the target data.

Security of the Optimal Lambda Prediction

This step is performed collaboratively among all participants, including the target client, source clients, and the aggregator. Throughout this process, the local data of the target client and the source clients are never directly exposed to either the aggregator or any other participant.

Aggregator's Perspective. During the federated training process, the aggregator receives masked model updates from the source clients, which it aggregates to compute the coefficients of the global model for the next iteration. The use of secure aggregation, implemented via zero-sum masking as formally proven in [1], ensures that a semi-honest aggregator cannot infer individual updates from any source client.

After the target client predicts the optimal lambda values, the aggregator receives these values. Since the target data is never directly shared, the aggregator cannot infer any information about the target data from the optimal lambda values.

Source Clients' Perspective. During the federated training process, source clients receive the updated global model after each training round. This global model consists only of model coefficients, ensuring that no information about other source clients' local data is exposed. Source clients never directly access one another's data, preserving the privacy of all local source data.

Once the target client predicts the optimal lambda values, the aggregator distributes these values to the source clients. Since the target data is never directly shared, source clients cannot infer any information about the target data from the received lambda values.

Target Client's Perspective. The target client receives only the coefficients of models trained using various regularization

parameters. These model coefficients are aggregate values that do not reveal any specific information about the local data of any source client.

Security of the Final Adaptive Model Training

This step is performed collaboratively among all participants, including the target client, source clients, and the aggregator. Throughout this process, the local data of the target client and the source clients are never directly exposed to either the aggregator or any other participant.

Aggregator's Perspective. The aggregator receives masked model parameters from the source clients, which it aggregates to compute the coefficients of the global model for the next iteration. The use of secure aggregation, implemented via zero-sum masking as formally proven in [1], ensures that a semi-honest aggregator cannot infer individual updates from any source client.

Source Clients' Perspective. During the federated training process, source clients receive the updated global model after each training round. This global model consists only of model coefficients, ensuring that no information about other source clients' local data is exposed. Source clients never directly access one another's data, preserving the privacy of all local source data.

Target Client's Perspective. The target client receives only the coefficients of models trained using various regularization parameters. These model coefficients are aggregate values that do not reveal any specific information about the local data of any source client.

References

1. Keith Bonawitz, Vladimir Ivanov, Ben Kreuter, Antonio Marcedone, H Brendan McMahan, Sarvar Patel, Daniel Ramage, Aaron Segal, and Karn Seth. Practical secure aggregation for federated learning on user-held data. *arXiv preprint arXiv:1611.04482*, 2016.
2. Ron Edgar, Michael Domrachev, and Alex E Lash. Gene expression omnibus: Ncbi gene expression and hybridization array data repository. *Nucleic acids research*, 30(1):207–210, 2002.
3. Anis Elgabli, Chaouki Ben Issaid, Amrit Singh Bedi, Ketan Rajawat, Mehdi Bennis, and Vaneet Aggarwal. Fednew: A communication-efficient and privacy-preserving newton-type method for federated learning. In *Proceedings of the International Conference on Machine Learning (ICML)*, 2022.
4. Lisa Handl, Adrin Jalali, Michael Scherer, Ralf Eggeling, and Nico Pfeifer. Weighted elastic net for unsupervised domain adaptation with application to age prediction from dna methylation data. *Bioinformatics*, 35(14):i154–i163, 2019.
5. Anika Hannemann, Ali Burak Ünal, Arjhun Swaminathan, Erik Buchmann, and Mete Akgün. A privacy-preserving framework for collaborative machine learning with kernel methods. In *2023 5th IEEE International Conference on Trust, Privacy and Security in Intelligent Systems and Applications (TPS-ISA)*, pages 82–90. IEEE, 2023.
6. Steve Horvath. Dna methylation age of human tissues and cell types. *Genome biology*, 14:1–20, 2013.
7. Peter Kairouz, Ziyu Liu, and Thomas Steinke. The distributed discrete gaussian mechanism for federated learning with secure aggregation. In *Proceedings of the International Conference on Machine Learning (ICML)*, 2021.
8. Jinhyun So, Ramy E. Ali, Başak Güler, Jiantao Jiao, and Salman A. Avestimehr. Securing secure aggregation: Mitigating multi-round privacy leakage in federated learning. In *Proceedings of the AAAI Conference on Artificial Intelligence*, 2023.
9. John N Weinstein, Eric A Collisson, Gordon B Mills, Kenna R Shaw, Brad A Ozenberger, Kyle Ellrott, Ilya Shmulevich, Chris Sander, and Joshua M Stuart. The cancer genome atlas pan-cancer analysis project. *Nature genetics*, 45(10):1113–1120, 2013.
